# Retraction: Cerebral microbleed detection via convolutional neural network and extreme learning machine

**DOI:** 10.3389/fncom.2023.1358283

**Published:** 2023-12-28

**Authors:** Frontiers Editorial Office

The journal retracts the 10 September 2021 article cited above.

Following publication, the publisher uncovered evidence that false identities were used in the peer-review process. The assignment of fake reviewers was confirmed by an investigation, conducted in accordance with Frontiers' policies and the Committee on Publication Ethics (COPE) guidelines. Given the concerns, the editors no longer have confidence in the findings presented in the article. UPDATE (30 July 2024): This notice is to alert readers of this matter, it does not imply involvement of the co-authors.

This retraction was approved by the Chief Editors of Frontiers in Computational Neuroscience and the Chief Executive Editor of Frontiers. The authors received a communication regarding the retraction and had a chance to respond. This communication has been recorded by the publisher.

